# Dynamics of urban green spaces in a megacity under the green economy framework and their influencing factors: a case study of Chongqing urban area

**DOI:** 10.3389/fpubh.2024.1517554

**Published:** 2025-01-15

**Authors:** Jing Ge, Yinghong Shi

**Affiliations:** College of Business Administration, Chongqing Vocational and Technical University of Mechatronics, Chongqing, China

**Keywords:** urban green space, urban public health, econometric models, economic drivers, urban green spaces evolution

## Abstract

**Introduction:**

In the context of escalating public health crises in megacities, promoting green and healthy urban spatial development is crucial. It not only contributes to economic growth and environmental sustainability but also significantly impacts the public health of urban residents.

**Methods:**

This study utilized land use data from 2000 to 2021 in Chongqing, China, to investigate the characteristics and patterns of change in urban green space distribution. Spatial analysis methods were employed, and an econometric model was constructed to analyze the mechanisms of these changes, considering factors such as economic drivers, governmental regulation, and social dynamics.

**Results:**

The results reveal that urban green spaces in the Chongqing metropolitan area are primarily concentrated in the inner suburbs with limited distribution in central urban areas, exhibiting a distinct spatial gradient. The overall size of urban green spaces has been decreasing, particularly in the inner suburbs. The primary patterns of this reduction include edge erosion in the inner suburbs, inward contraction in central urban areas, and minor perforation in new towns.

**Discussion:**

The analysis indicates that economic drivers, such as industrial development and economic growth, are the dominant factors contributing to the reduction of urban green spaces. The impact of social dynamics, such as public demand, appears to be relatively insignificant. Conversely, government planning policies and public investments play a crucial role in the protection and development of urban green spaces. These findings emphasize the need for effective urban planning strategies that prioritize the conservation and expansion of green spaces to enhance public health and environmental sustainability in megacities.

## 1 Introduction

With economic development and rapid urbanization, residents are increasingly striving for improved quality of life, higher leisure expectations, and greater environmental awareness, which has led to a rising demand for urban green spaces. Research related to urban green spaces in megacities is becoming increasingly important (1, 2). Urban green spaces, characterized by their life-supporting, beautifying, and social service functions, are natural or semi-artificial ecosystems that involve human interaction. These spaces not only enhance the ecological environment and optimize urban landscapes, but also promote physical activities among residents, alleviate mental stress, and reduce negative emotions (3–6), playing a crucial role in improving urban living conditions and enhancing the satisfaction, happiness, and well-being of city dwellers (7). In addition, urban green spaces cater to emergency needs during disasters such as earthquakes (8), and they also play a critical role in the dilution of pollutants (9). These functions highlight the importance of integrating green spaces into urban planning, as they not only enhance the quality of life for residents but also contribute to the resilience and sustainability of cities in the face of environmental challenges.

Urban green spaces differ from traditional urban green spaces; they form a green network system comprised of gardens, urban forests, vertical greening, urban farmlands, and aquatic wetlands (10, 11). They serve multiple functions such as climate regulation, environmental purification, biodiversity maintenance, and the improvement of public health and leisure facilities (12, 13), making them a vital component of urban spaces. Urban green spaces are significant for human health and welfare. However, due to their increasing importance and scarcity, the distribution of urban green spaces often varies with the socioeconomic status (SES) of residents, leading to disparities in the health benefits enjoyed by different social groups. This results in” green health” inequalities between different socioeconomic statuses (14–16).

Existing research primarily focuses on the critical role of urban public services in public health, with limited econometric models developed to analyze the evolution mechanisms of urban green spaces from perspectives such as economic drivers, government regulation, and social dynamics. However, studies on the dynamic evolution of urban green space patterns are already quite extensive and mainly concentrate on the following aspects: (1) Study of urban green space scale and structure. The spatiotemporal dynamics of scale and structure are foundational to quantitative research on urban green space patterns (17–20), often based on land use data and employing statistical methods to quantitatively analyze urban green spaces and their components (10, 21), or using concentric zoning to reveal gradient distribution patterns or employing metrics like dynamism and change intensity for quantitative dynamic analysis (17, 22, 23). (2) Urban green space conversion research. This typically utilizes the transition matrix model on the ArcGIS platform to measure and visualize land use conversions (24–26), further analyzing the spatiotemporal characteristics of urban green space transformations. (3) Analysis of urban green space landscape indices. Landscape indices condense information about landscape patterns, often quantitatively reflecting the spatial structure and configuration by selecting indices related to patches, types, and landscape levels (25–29). However, relying solely on index comparisons can fail to account for the complex characteristics of changes, which is a limitation of this approach.

Overall, contemporary research on urban green space pattern changes tends to focus on explicit spatial representations such as scale, distribution, and landscape, but studies on the patterns of evolution of urban green spaces, i.e., how urban green spaces evolve, are less common (10, 22, 23, 30). This lack of research makes it difficult to accurately assess the processes of urban green space change and their interactions with constructed spaces, which is detrimental to the planning, construction, and optimization of urban green spaces. Moreover, there is generally less research on the driving mechanisms behind changes in urban green space patterns, with the few existing studies mostly providing qualitative analyses of influencing factors or combining qualitative and quantitative analyses based on factor analysis (17, 31–35). Comprehensive dissection and quantitative research on the intrinsic driving mechanisms are urgently needed.

This study focuses on Chongqing, a megacity with a central role in the Chengdu-Chongqing urban agglomeration, employing econometric models to analyze the regulation and driving mechanisms behind the evolution of urban green spaces, as well as examining their transformation during the urbanization process and their impact on the ecological environment. By analyzing land use data from 2000 to 2021, this paper reveals the basic scale, distribution, and spatiotemporal evolution characteristics of urban green spaces in Chongqing. The research utilizes GIS spatial analysis and mathematical-statistical methods to delve into how urban expansion affects urban green spaces, particularly how the expansion of construction land encroaches on green areas (32, 36). The results indicate that as the city expands into peripheral areas, the urban green spaces in Chongqing are significantly compressed, especially where suburban green areas are converted into construction sites most severely. Although the urban green spaces in the central urban areas have received some attention and have increased, the new green areas are mainly limited to parks and roadside green belts. Additionally, by applying spatial econometric models, this study also explores the driving mechanisms behind the evolution of urban green spaces, providing a scientific basis for urban planning and management. The findings not only offer strategic recommendations for optimizing urban green spaces and regional sustainable development in Chongqing but also serve as a reference for urban planning and urban green space management in other developed regions. By integrating urban green space planning into the overall urban planning framework, it can promote rational, scientific, and sustainable development of cities, effectively mitigating the conflicts between ecological environment and economic growth during urbanization.

## 2 Study area

**Figure 1:**
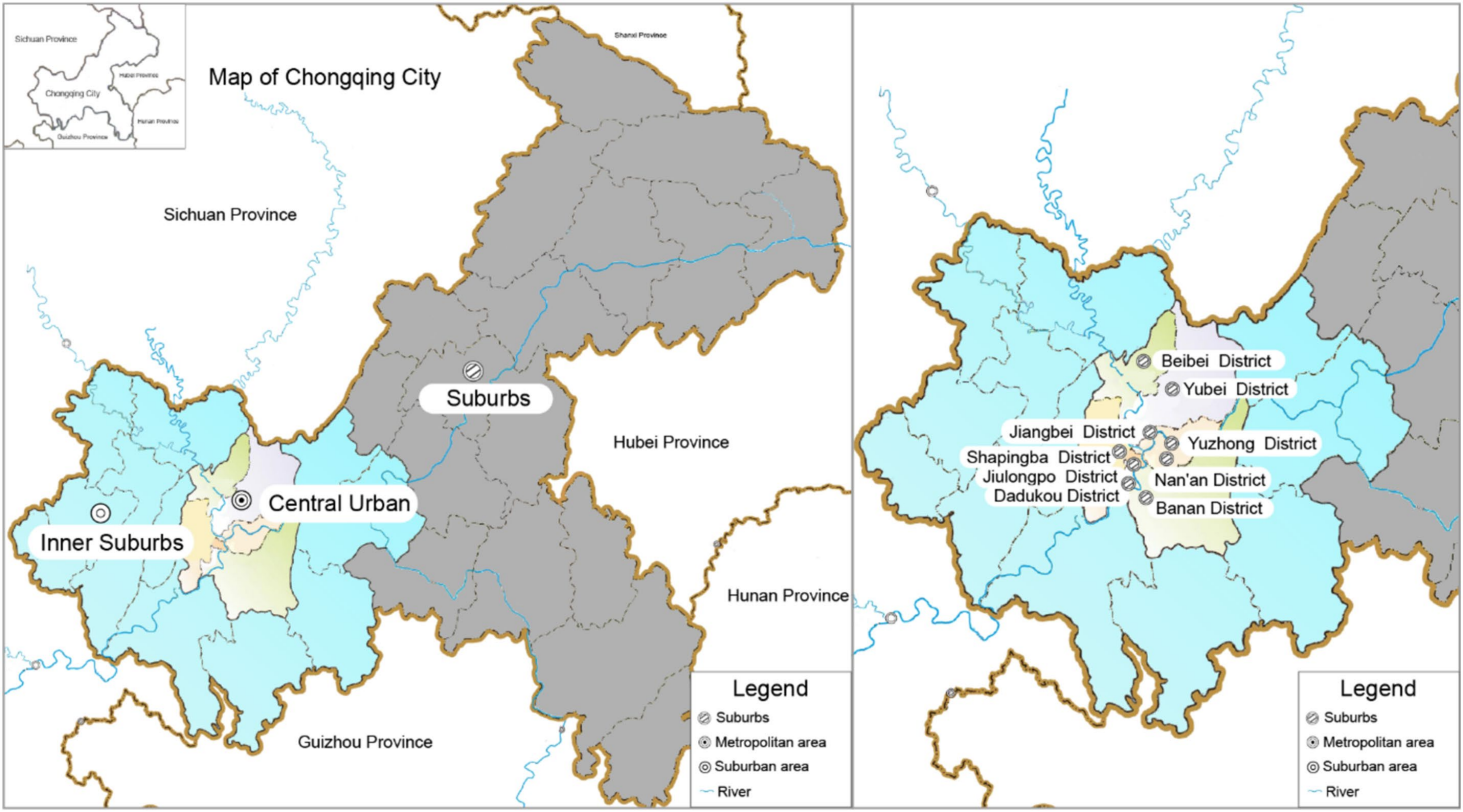
Evolution of urban green space patterns. Geographical locations and zonal stratification of central urban and inner suburbs in Chongqing.

Chongqing, a direct-controlled municipality in the People’s Republic of China, stands out for its dynamic urban environment, complex transportation systems, and large population, as shown in Figure 1. As a major urban center in southwestern China, Chongqing has experienced rapid urbanization and population growth, reflecting broader trends in Chinese metropolitan development (37).

Chongqing’s urban environment is characterized by a unique blend of modern high-rise buildings and traditional Chinese architecture, set against a backdrop of natural scenery that includes mountains and rivers. The city’s topography, with its many hills and slopes, has influenced the distinctive layout and spatial organization of urban areas. Chongqing is also known for its significant urban green spaces within the city limits, which help mitigate the urban heat island effect, although these areas are under pressure from ongoing urban expansion.

The city struggles with substantial environmental challenges, notably air and water pollution. Industrial activities, along with heavy vehicular traffic, contribute to high levels of air pollutants. Efforts to improve air quality are ongoing and include measures to reduce industrial emissions and increase the use of public transportation. Additionally, Chongqing faces issues with water quality due to industrial waste and urban runoff affecting its major waterways, prompting several government-led initiatives aimed at water conservation and treatment (5, 13).

With a population exceeding 30 million people in its administrative area, Chongqing is one of the most populous cities in China. The population density varies significantly across the municipality, with dense urban centers and more sparsely populated rural districts. The demographic composition reflects a mix of the Han majority and various ethnic minorities, including the Tujia, Miao, and others, contributing to the city’s cultural diversity.

Chongqing’s demographic trends are influenced by both rural-to-urban migration and the government’s policies promoting urbanization. This migration has fueled urban growth but also poses challenges in terms of service provision, housing, and social integration for new urban residents.

## 3 Methods

This research builds on the hierarchical structuring of the Chongqing metropolitan area and utilizes spatial analysis techniques to examine the scale, spatial distribution gradients, and temporal dynamics of urban green spaces within Chongqing (19, 33, 38). Based on this analysis, the study develops a theoretical model consisting of three dimensions’: economic drivers (20, 30, 39, 40), social dynamics (26, 41, 42), and governmental regulation (17, 34, 43, 44). This model is designed to thoroughly assess and identify the principal factors driving the changes in urban green spaces.

By selecting pertinent driving factors, this research utilizes spatial econometric models (30, 31) to thoroughly dissect the dynamic mechanisms of urban green space development and change, revealing how economic growth, social development, and policy interventions collectively influence the layout and functional evolution of urban green spaces. This not only aids in understanding the role of urban green spaces within urban ecosystems but also provides scientific decision-making support for urban planners to foster a harmonious coexistence between economic development and environmental protection.

### 3.1 Spatial econometric model

To reveal the spatiotemporal characteristics of urban green space distribution and to provide scientific decision support for urban greening management and planning, as well as to explore its application value in urban expansion, ecological protection, and sustainable development, we conducted a hot and cold spot analysis of urban green spaces within the study area. This analysis deeply investigates the spatial clustering characteristics of urban green space changes and their distribution differences across various urban tiers. The transformation of urban green spaces has become increasingly prominent due to rapid urbanization, population growth, and economic development. These changes are influenced by multiple factors, including urban planning policies, land-use patterns, socioeconomic conditions, and environmental protection initiatives. Understanding the gradient variations in green spaces is crucial as they reflect the complex interactions between urban development and ecological preservation. The spatial distribution patterns of green spaces not only indicate the current state of urban ecological systems but also serve as important indicators for evaluating urban sustainability and environmental quality. Furthermore, these patterns provide valuable insights into the effectiveness of urban planning strategies and highlight areas requiring enhanced environmental protection measures.

Hot and cold spot analysis is an efficient spatial data analysis technique that quantifies and identifies local spatial association characteristics using the Getis-Ord $G_{i}^{*}$ index, enabling the manifestation of statistically significant hot spots (high-value clusters) and cold spots (low-value clusters). In this analytical model, hot spot regions represent clusters significantly above the overall average, while cold spot regions indicate statistically significantly lower than average values.

For precise assessment and analysis, this study designed a 2 km by 2 km grid as the evaluation unit and selected the change in urban green spaces within different periods as the main research indicators. Using ArcGIS software, we calculated the local spatial associations of each evaluation unit based on the Getis-Ord $G_{i}^{*}$ index (45). Further, we used the Jenks natural breaks method (46) to categorize the results into four levels: hot spots, sub-hot spots, sub-cold spots, and cold spots, to analyze in detail the evolution of urban green space patterns and their hot and cold spot changes. The specific computation formula is as follows:


$$G_{i}^{*}\left(d\right)=\frac{\sum_{j}^{n}W_{ij}\left(d\right)X_{j}}{\sum_{j}^{n}X_{j}}$$

To make the analysis results more comparative and interpretable, the following normalization was performed:


$$Z\left(G_{i}^{*}\right)=\frac{G_{i}^{*}-E\left(G_{i}^{*}\right)}{\sqrt{Var\left(G_{i}^{*}\right)}}$$

where $E\left(G_{i}^{*}\right)$ and $Var\left(G_{i}^{*}\right)$ are the expected value and variance of $G_{i}^{*}$ , respectively, and $W_{ij}\left(d\right)$ represents spatial weights. If $Z\left(G_{i}^{*}\right)$ is positive and statistically significant, it indicates that the values around location i are relatively high (above average), belonging to a high-value spatial cluster (hot spot area); conversely, if $Z\left(G_{i}^{*}\right)$ is negative and statistically significant, it suggests that the values around location i are relatively low (below average), belonging to a low-value spatial cluster (cold spot area).

### 3.2 Patterns changing in urban green spaces evolution

The expansion of construction land is the direct cause of changes in the proportion of urban green spaces, which exhibits different spatial expansion patterns under the influence of various factors. Typically, these patterns can be categorized into three main types: infill, sprawl, and exclave.

The infill expansion pattern occurs when new construction fills in the existing gaps within urban areas, leading to the densification of built-up areas. This pattern is characterized by the efficient utilization of urban land resources and typically results in the reduction of scattered green spaces within the urban core.

The sprawl expansion pattern manifests in two distinct forms: radial and axial expansion. Radial expansion involves urban growth extending outward from the city center in all directions, creating a concentric pattern of development. This pattern often results in the gradual consumption of peripheral green spaces. Axial expansion, on the other hand, occurs along major transportation corridors or development axes, forming linear patterns of urban growth that can fragment existing green spaces along these routes.

The exclave expansion pattern is characterized by the development of isolated construction areas separated from the main urban body. This pattern often creates discontinuous urban landscapes and can lead to the fragmentation of larger green space patches.

**Figure 2:**
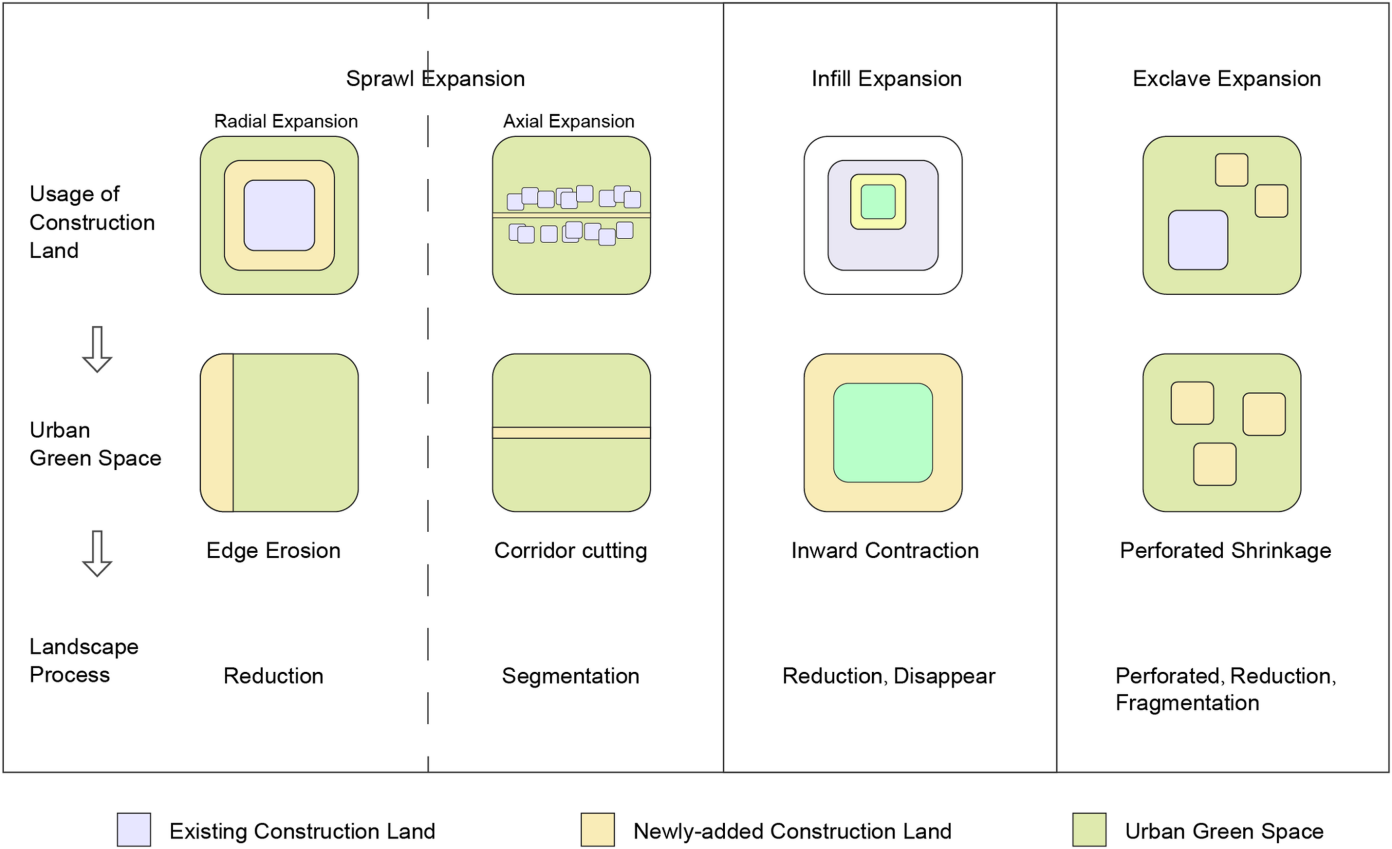
Evolution of urban green space patterns.

Based on the interaction between construction land and urban green spaces, the evolutionary patterns of urban green space contraction can be summarized into four types: edge erosion, corridor cutting, inward contraction, and perforated shrinkage, as illustrated in Figure 2. Edge erosion occurs when urban development gradually encroaches upon the periphery of green spaces. Corridor cutting refers to the fragmentation of green corridors by new construction. Inward contraction describes the process where green spaces shrink from their outer boundaries toward their centers. Perforated shrinkage occurs when small patches of development appear within larger green spaces, creating a perforated pattern.

In terms of specific research methods, the study first identifies the expansion patterns of construction land by extracting newly added construction patches from different periods and applying the minimum bounding rectangle method and convex hull model method to comprehensively determine the spatial structure and boundaries of the expansion. These methods effectively reveal the spatial structure and boundaries of the expansion of construction land. Subsequently, using the Erase tool on the ArcGIS platform, the study precisely extracts the newly added and contracted urban green spaces of construction units in Chongqing over different periods, which is key to understanding the dynamic changes of urban green spaces. Finally, by overlaying the evolutionary patterns of urban green spaces with the corresponding periods’ construction land evolution patterns, the study detailedly analyses the spatiotemporal evolution characteristics of urban green spaces.

Through the above analysis, this study not only provides a scientific methodological model to observe and assess the evolutionary trends of urban green spaces but also offers data support and theoretical basis for the formulation of urban planning and sustainable development policies. The application of this method contributes to optimizing urban spatial structure, enhancing urban ecological quality, thereby better achieving the goals of sustainable urban development.

### 3.3 Evolution mechanisms

#### 3.3.1 Interaction factors

**Figure 3:**
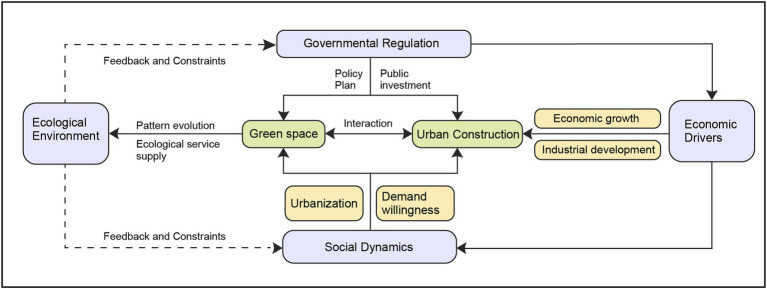
Analysis framework of urban green space patterns evolution.

The intricate interplay between socio-economic drivers, social growth forces, and governmental control mechanisms significantly shapes the evolution of urban spaces, particularly urban green spaces. This dynamic relationship involves a series of interactions that not only influence the spatial configuration but also the functional quality of urban environments (Figure 3).

Economic drivers play a foundational role in urban spatial dynamics. As regional GDP grows, there is an increase in residents’ disposable incomes and a corresponding rise in investments in urban infrastructure and real estate. This economic growth leads to a proliferation of urban construction, which can encroach upon green spaces unless carefully managed. The expansion of sectors such as real estate directly impacts the availability and distribution of urban green spaces. Moreover, the development of industrial clusters and parks further intensifies the demand for urban land, potentially at the expense of green spaces. These economic activities necessitate a strategic approach to urban planning that integrates green space conservation amidst economic development.

Social dynamics, encompassing community organizations, NGOs, and the general populace, play a crucial role in shaping urban green space policies. The preferences of urban residents, their participation in community movements, and their engagement in environmental activities influence urban developers and policymakers. For instance, a strong demand for green spaces can lead developers to prioritize these areas within their projects. Moreover, the actions of NGOs and community groups can lead to increased public awareness and pressure on governmental bodies to enact and enforce policies that protect and enhance urban green spaces. The social dynamic, therefore, acts as a vital feedback mechanism that informs and sometimes challenges governmental and economic priorities.

Governmental regulation serves as the overarching framework that guides the evolution of urban land use, including green spaces. By implementing laws, regulations, and urban planning policies, the government can ensure that green spaces are preserved and integrated into the urban fabric, even as the city expands and evolves economically. Strategic urban planning, supported by regulations and policies, can mitigate the adverse effects of unchecked urban sprawl and economic development on green spaces. Furthermore, government investments in the development of various types of urban green spaces—public, ancillary, ecological protective, and productive—demonstrate the role of governmental agencies in actively shaping the urban landscape in favor of sustainable and livable urban environments.

The interaction among these three drivers—economic, social, and governmental—creates a complex system where changes in one aspect can have ripple effects across others. Economic growth can lead to more robust social dynamics as communities become more empowered and financially capable to demand sustainable urban environments. Conversely, strong social dynamics can influence governmental policies, which in turn can regulate economic activities to ensure sustainable urban development. The mechanisms of feedback and adaptation among these forces are essential for the sustainable management of urban green spaces. Policies must be adaptive and responsive, incorporating feedback from economic data and social demands. This adaptive approach ensures that urban green spaces are not only preserved but are also functionally integrated into the broader urban ecosystem, contributing to the overall health, sustainability, and resilience of urban areas. Understanding and harnessing these interactions is crucial for city planners, policymakers, and community leaders aiming to create sustainable urban environments that balance ecological, economic, and social needs effectively.

#### 3.3.2 Metrics

In this study, 245 streets of Chongqing City were selected as the research units. Given the availability of data, we selected relevant indicators from three dimensions: economic drivers, social dynamics, and governmental regulation. Using SPSS statistical software, we performed a collinearity analysis on the chosen indicators to identify and eliminate variables with strong collinearity. Specifically, the variance inflation factor (VIF) (47) was calculated for each factor to assess the degree of collinearity, and factors with a VIF value exceeding 10 were progressively excluded to ensure the independence of the remaining variables. This method helps to enhance the accuracy and explanatory power of the model.

Details of the selected indicators are presented in Figure 3. This selection process not only strengthened the robustness of the model’s predictions but also ensured the scientific validity and reliability of the analysis results. Through this rigorous data processing and analysis procedure, we ensured that the study results accurately reflect the real impact of economic drivers, social dynamics, and governmental regulation on the evolution of the urban spatial structure at the street level in Chongqing City.

#### 3.3.3 Model construction

In the empirical research on the drivers behind the evolution of urban green space patterns, commonly used statistical models include Ordinary Least Squares Regression (OLS) (48), Spatial Lag Model (SLM) (49), and Spatial Error Model (SEM) (50). The spatial evolution of urban green space patterns not only exhibits significant spatial autocorrelation but also requires mathematical models to precisely express its geographical spatial structure and define the adjacency relationships between research subjects. However, traditional OLS models are not suitable for analyzing the correlation of spatial data (33, 48), therefore, spatial weight matrices are introduced and SLM and SEM models are employed to more accurately describe the spatial relationships between the dependent and independent variables.

1. The Spatial Lag Model (SLM) is primarily used to examine the spatial spillover effects of the dependent variable within the study area, exploring the spatial diffusion phenomena of environmental or socio-economic activities (4). The SLM is capable of effectively capturing and revealing the interactions and mutual influences between spatial units. The model expression is as follows:

$$y=\rho W_{y}+X\beta +\varepsilon$$

Here, X represents the n×k matrix of explanatory variables; W is the n×n spatial weight matrix, used to describe the interactions between spatial units; ρ is the spatial autoregressive coefficient, reflecting the degree of spatial diffusion or spillover; $W_{y}$ represents the spatially lagged variable, indicating the influence of the dependent variable in neighboring areas; β are the coefficients of the explanatory variables on the dependent variable y ; ε is the random error term.

1. The Spatial Error Model (SEM) is primarily utilized to address the spatial correlation among model error terms caused by measurement errors or omitted variables. The SEM can accurately assess the direction and degree of the impact of neighboring areas’ error terms on the observed values of the dependent variable. The model expression is as follows:

$$y=X\beta +\lambda W_{\mu }+\varepsilon$$

In this equation, y is the dependent variable; X represents the n×k matrix of independent variables, where n is the number of samples and k is the number of independent variables; W is the n×n spatial weight matrix, used to reflect the spatial trends of the independent variables; μ represents the random error vector; λ is the spatial autocorrelation coefficient, used to measure the spatial dependency among samples; ε is a normally distributed random error vector.

The application of these two spatial statistical models allows for a deeper understanding and analysis of the spatial dynamics and driving mechanisms behind the evolution of urban green space patterns.

### 3.4 Data collection

This study primarily utilized Landsat TM remote sensing imagery data from four periods: 2000, 2007, 2014, and 2021, for the Chongqing area. The dataset includes raster data of vegetation Net Primary Productivity (NPP) and Quality Control (QC) percentages at a 500-meter resolution for the Yangtze River Economic Belt from 2000 to 2021. These data were extracted from the MOD17A3HGF.v006 dataset released by NASA based on the 1:250,000 scale vector boundaries of the Yangtze River Economic Belt, and stored in the TIFF format.

In terms of land use classification, this study referred to the International Geosphere-Biosphere Programme (IGBP) Land Use and Land Cover Change (LUCC) classification system, adjusted according to the actual conditions and research needs of Chongqing, categorizing land use into five main types: farmland, forest land, grassland, water bodies, and built-up land. Among these, farmland, forest land, grassland, and water bodies were classified as urban green space land.

Additionally, the socioeconomic data involved mainly came from the Chongqing Statistical Yearbook of the corresponding years and some statistical materials published by district governments. To eliminate the effects of time-related price fluctuations and changes in monetary value, the data were adjusted using the price index. For some missing data, estimates and supplements were made according to the proportion of the population of each street or district to the total regional population. Due to administrative division adjustments, data from 2000 onwards have been adjusted and revised according to the new district divisions established after the abolition of counties.

Policy variables were designed as dummy variables, assigned based on a comprehensive assessment of the implementation degree of policies related to urban green spaces, marking each evaluation unit as 0 or 1 to represent whether the policy was implemented or not. This method effectively quantified policy impact factors, facilitating the analysis of their driving effects on changes in urban green spaces in subsequent models. Through the collection and organization of this series of data, this study aims to deeply explore and understand the evolution and driving mechanisms of the urban green space pattern in the Chongqing area.

## 4 Results

### 4.1 Overall changes in the scale of urban green spaces

From Table 1, between 2000 and 2021, the total area of urban green spaces in Chongqing exhibited a marked decreasing trend. Specifically, the area of urban green spaces reduced from 3580.97 square kilometers in 2000 to 2959.30 square kilometers in 2021, showing a trend of acceleration followed by a deceleration in reduction. Breaking down the periods, from 2000 to 2007, urban green spaces decreased by 175.41 square kilometers, with an annual average reduction rate of approximately 0.98%; entering the 2007 to 2014 phase, as new urban constructions and industrial parks rapidly developed, the encroachment on urban green spaces became more severe, with the annual average reduction rate increasing to 1.87%; whereas from 2014 to 2021, the rate of decrease in urban green spaces slowed, with a reduction of 127.07 square kilometers, and the annual average reduction rate dropped to 0.82%.

**Table 1:** Temporal variations in the scale of green spaces in Chongqing.

Types	2000–2007		2007–2014		2014–2021	
	Variation/km^2^	Rate%	Variation/km^2^	Rate%	Variation/km^2^	Rate%
Forest	−14.10	−3.01	18.15	4.45	89.64	21.12
Grassland	4.24	8.21	0.46	0.09	13.45	16.45
Water area	−37.81	−7.81	43.79	10.64	−21.41	−9.64
Urban green space	−201.21	−4.1	−679.12	−12.6	−145.15	−3.14

Looking at the internal composition changes of urban green spaces, the overall reduction was mainly due to the increase in forest lands, grasslands, and water bodies, coupled with the continuous decrease in farmland areas. While Chongqing has implemented various protective measures, several factors have limited their effectiveness: (1) Imbalanced Protection Strategy: The city’s focus on urban greening and wetland restoration, while commendable, has been primarily concentrated in specific areas, leaving other regions vulnerable to development pressure. (2) Resource Allocation Issues: Despite increased investment in ecological restoration, the resources allocated have been insufficient to counter the rapid pace of urbanization and industrial development. (3) Policy Implementation Gaps: Though environmental protection policies exist, their enforcement has been challenging due to competing economic development priorities and insufficient coordination among different governmental departments. (4) Limited Compensation Mechanisms: The lack of effective compensation mechanisms for farmland protection has accelerated the conversion of agricultural land to other uses.

Despite Chongqing’s vigorous efforts in recent years to promote urban greening, dredging of lakes and water bodies, and ecological restoration of the Yangtze River wetlands, which have yielded certain achievements, the positive impacts of these measures have still been insufficient to completely offset the overall trend of reduction in urban green space area caused by the significant decrease in farmland.

Thus, it is evident that Chongqing faces a contradiction between the protection of urban green spaces and land development utilization in the process of urbanization. The city’s current approach, while showing some positive results, has been inadequate in addressing the fundamental challenges of balancing urban development with environmental protection. This situation calls for a more comprehensive and integrated approach to urban planning and environmental conservation, incorporating stronger protective measures for farmland, more effective enforcement mechanisms, and better coordination between development and conservation goals.

### 4.2 Changes in the spatial gradient of urban green spaces

**Figure 4:**
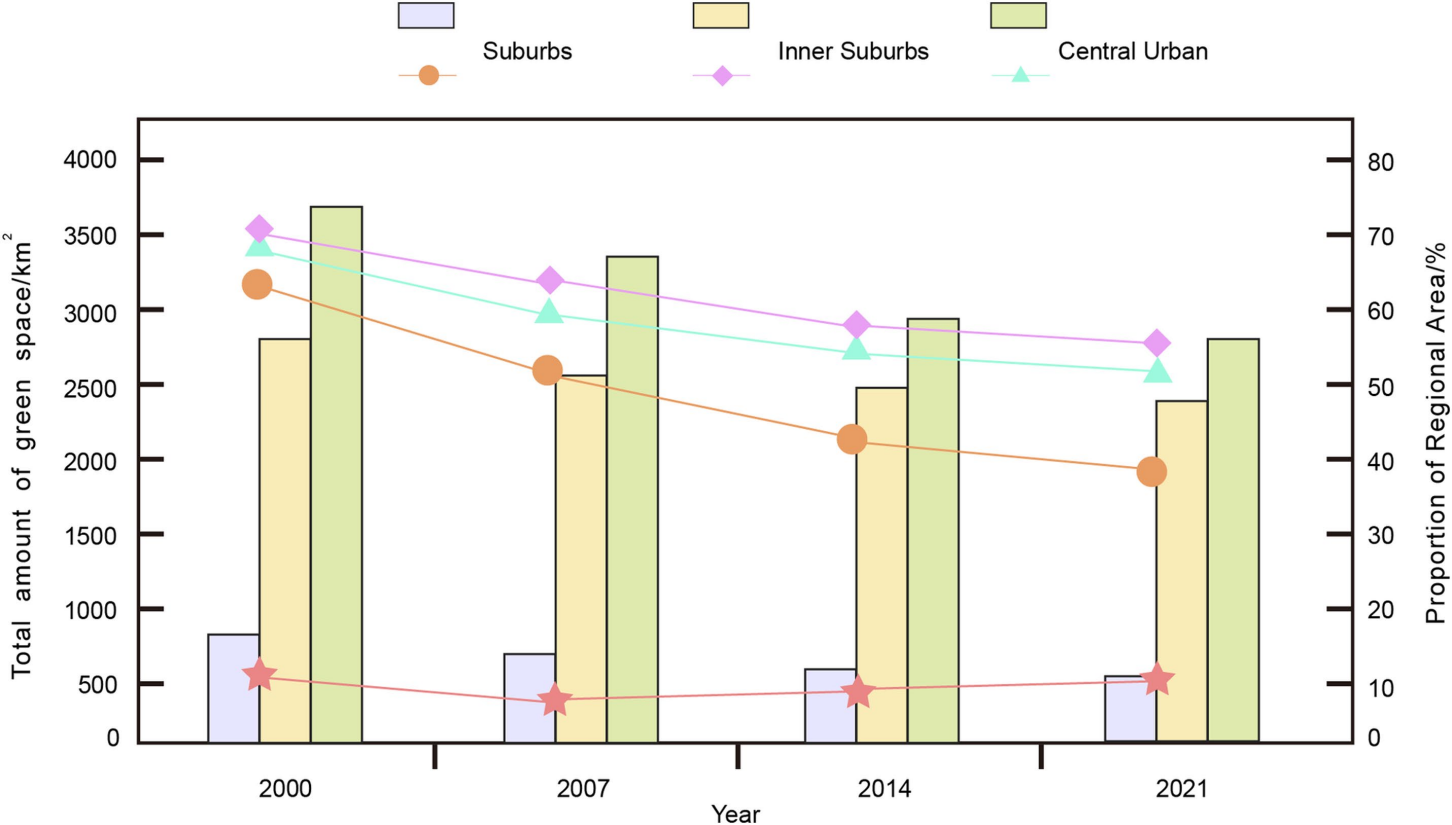
Changes in the area and proportional allocation of urban green spaces in Chongqing.

The scale of urban green spaces in Chongqing City exhibits distinct gradient changes, expanding progressively from the central urban area to the inner suburbs and suburbs, where the area shows a significant increasing trend. According to 2021 data, the areas of urban green spaces in these three major regions account for 12.02, 47.26, and 67.24% of their respective geographic units, as shown in Figure 4. The spatial distribution of urban green spaces in Chongqing shows a distinct gradient pattern from the central urban area to the suburbs. As of 2021, green space coverage accounts for 12.02, 47.26, and 67.24% in the central urban area, inner suburbs, and suburbs, respectively. The central urban area, dominated by high-density development, shows the lowest but relatively stable green space proportion with an annual decline of 0.38%. The inner suburbs, serving as a transitional zone, experience the most dramatic reduction (2.30% annually) due to rapid urbanization and industrial development. In contrast, the suburbs maintain the highest green coverage and demonstrate the most stability with only a 1.00% annual decline, primarily due to their role in ecological conservation. This gradient pattern reflects the varying intensities of urban development pressure and the effectiveness of environmental protection measures across different urban zones.

**Figure 5:**
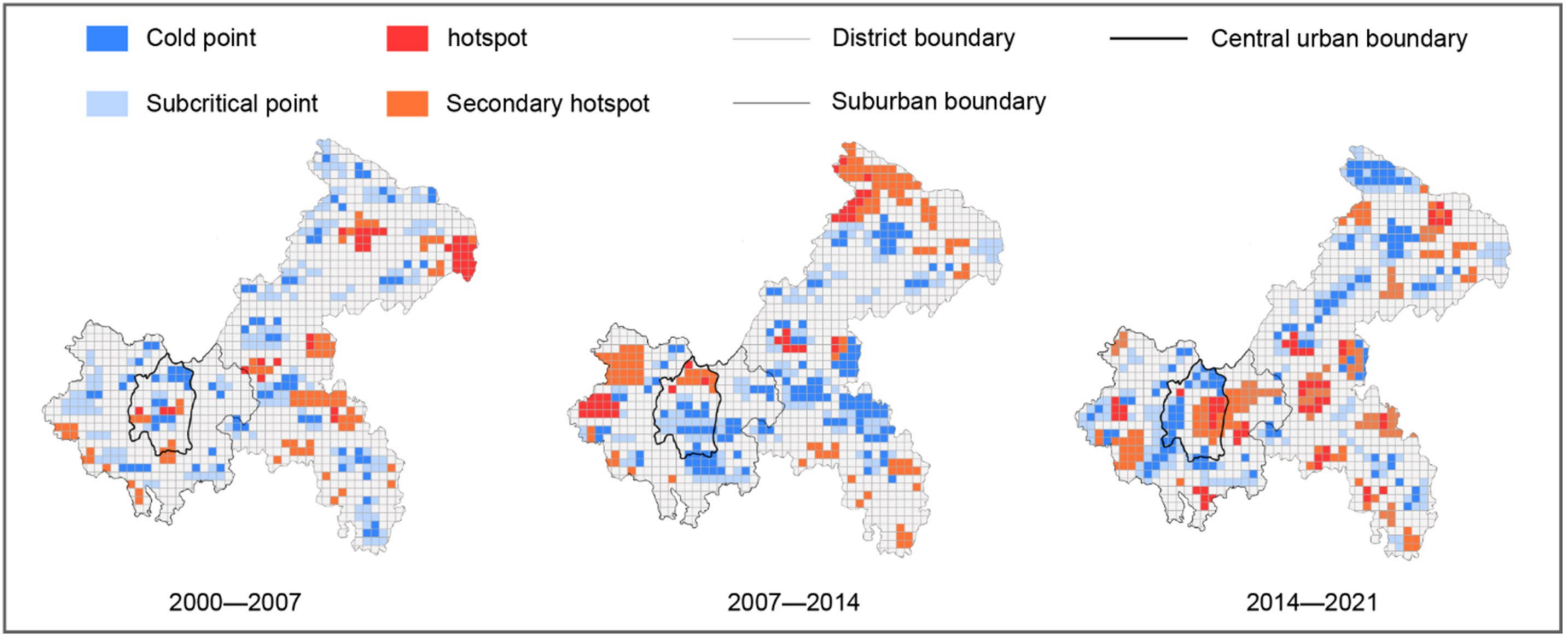
Evolution of hot and cold spots in the urban green space patterns of Chongqing.

From Figure 5, through hot spot and cold spot analysis, it is evident that from 2000 to 2021, the number and coverage area of hot spots and cold spots of urban green spaces in Chongqing have significantly increased. Hot spots mainly shift from the periphery to the central urban, reflecting the gradual formation of urban expansion and a polycentric development model. This shift indicates successful implementation of urban greening policies and suggests improved ecological network connectivity, potentially enhancing urban microclimate regulation and biodiversity conservation while increasing property values in surrounding areas.

Meanwhile, cold spots spread from the center to the periphery, particularly in the central urban and inner suburbs areas, where a large amount of urban green space is encroached upon by new construction projects. This pattern signals concerning trends including increased fragmentation of existing green spaces, disruption of ecological corridors, and reduced ecosystem service capacity. Such changes may lead to heightened urban heat island effects, compromised urban ecological security, and diminished recreational spaces.

These spatial evolution patterns highlight the need for more balanced urban development strategies that strengthen the protection of remaining green spaces while supporting urban growth. This calls for the implementation of comprehensive green infrastructure networks, development of compensatory mechanisms for lost green spaces, and better integration of ecological considerations in urban expansion planning to ensure long-term environmental sustainability.

From 2000 to 2007, there were fewer hot spot areas, primarily concentrated in central and inner suburbs areas such as Yuzhong District, Jiulongpo District, Jiangbei District, and Nan’an District, with notable green belt construction along highways and major roads. Conversely, cold spots were mainly concentrated in more distant areas such as Jiangjin District and Nanchuan District.

As the period from 2007 to 2014 progressed, the importance of urban green spaces in the central urban area gradually increased, with hot spots and secondary hot spots beginning to appear in main urban districts such as Yuzhong, Jiangbei, and Nan’an, and gradually expanding to areas like Nanchuan District and Bishan District, as shown in Table 1. During this stage, urban green space construction was mainly focused along the green belts and ecological corridors beside the Jialing River and Yangtze River. Concurrently, cold spots gradually shifted from the main urban districts and concentrated mainly in newly established sub-cities and new towns such as Beibei District, Shapingba District, Yubei District, and in Bishan District, Nanchuan District, Changshou District, Jiangjin District, reflecting the potential constraints of new urban construction on the growth of urban green spaces.

Entering the period from 2014 to 2021, with the further advancement of urban ecological civilization construction, new cities and towns in central urban areas and their suburbs became important regions for the increase of urban green spaces, while areas along the Yangtze River and in the suburbs, especially the northern areas of the suburbs, became the main cold spots, demonstrating a dynamic balance and adjustment between urban development and ecological protection.

This evolution has significantly impacted urban ecosystems through water regulation, microclimate modification, and biodiversity support, while facing challenges from continuous urban expansion. The patterns highlight the need for comprehensive green infrastructure planning and effective conservation strategies. As cities continue to develop, maintaining balance between urban growth and ecological preservation remains crucial, requiring adaptive management approaches and long-term protection mechanisms to ensure sustainable urban development while preserving essential ecosystem services.

### 4.3 Influencing factors

Using GeoDa software, this study estimated and tested the Spatial Error Model (SEM) and the Spatial Lag Model (SLM), as shown in Table 2. The SEM primarily addresses spatial autocorrelation in observational errors, specifically designed for scenarios where error structures exhibit spatial dependencies. The basic form of SEM is expressed as y=Xβ+u , where u=λWu+∈ represents the error term, with spatial structure characterized by the spatial weight matrix W and autocorrelation coefficient λ . In contrast, SLM focuses on capturing spatial correlations among dependent variables, formulated as y=ρWy+Xβ+∈ , where ρ denotes the spatial lag coefficient indicating the inter-regional influence of dependent variables. This model is particularly effective in scenarios with significant regional interactions.

**Table 2:** Temporal variations in the scale of green spaces in Chongqing.

Model	Lagrange multiplier	Robust LM	R2	LR	AIC	SC
SLM	221.1682	1.342	0.54783	357.1241	3454.14	3647.13
SEM	286.4311	41.167	0.58731	434.4671	3647.46	3476.12

The Lagrange Multiplier test for error (LM-error) in the spatial error model was significantly more robust than that for the spatial lag model (LM-lag), aligning with the model selection criteria proposed by Pham et al. (38), indicating the appropriateness of the spatial error model. In terms of model fit, the SEM demonstrated superiority over the SLM, as evidenced by a higher Likelihood Ratio (LR) and lower Akaike Information Criterion (AIC) and Schwarz Criterion (SC) (39, 40, 47). Therefore, this study chose the spatial error model for regression analysis of the evolution of urban green spaces in Chongqing City.

1. In terms of economic drivers of the evolution of urban green space patterns, the proportion of the tertiary sector and the area of industrial parks significantly and negatively influenced urban green spaces in Chongqing City, particularly the construction of industrial parks, which had a significant negative impact at the 0.1 level.

Specifically, a *p*-value of 0.1 indicates a 10% probability that the observed effect in the data occurred by chance, while a value of 0.01 represents a 1% probability of such random occurrence. In computational research and data analysis, lower *p*-values (such as 0.05 or below) are generally considered to demonstrate strong statistical significance. Therefore, the interpretation of these values is crucial for assessing the reliability and validity of research findings. This statistical threshold serves as a quantitative metric for evaluating the robustness of experimental results and helps researchers determine whether their findings represent genuine patterns or random variations in the data.

As Chongqing’s industrial structure continued to optimize and adjust, the driving force behind urban land use restructuring increased. The tertiary sector mainly concentrated in the central urban areas, while the secondary sector shifted to new cities and suburbs like Banan New City, Yubei New City, Nanchuan District, and Changshou District. The construction of multiple industrial parks and economic and technological development zones in suburban areas led to extensive land occupation, eroding urban green spaces and becoming a cold spot area for change. Economic growth had both positive and negative impacts on urban green spaces at different levels, where regional GDP had a significant positive effect on urban green spaces at the 0.05 level. High GDP regions, such as the main city and other central areas, which have higher urban construction levels, paid more attention to urban greening. Financial investments in green infrastructure and maintenance facilitated the management of urban green spaces, promoting the construction of urban green spaces and turning these areas into hot spots for change. Real estate investment, although negatively impacting urban green spaces, was not significant at conventional levels.

1. In terms of social growth forces, including per capita disposable income of urban residents and proximity to urban green spaces, the driving effects on Chongqing’s urban green spaces were not significant. The urban permanent population had a significant negative impact on urban green spaces, with a coefficient of −0.6027 at the 0.01 level. As urbanization progressed, the growth and aggregation of the population intensified the demand for residential and living spaces, leading to an increase in the proportion of residential and related infrastructure lands, thereby encroaching on urban green spaces. From 2000 to 2021, suburban areas like Banan District, Jiangjin District, Changshou District, and Bishan District, which experienced significant economic development and population aggregation, saw an expansion in construction lands, becoming key areas where urban green spaces were eroded.
2. In terms of governmental control, planning policies had a significant positive impact on the urban green spaces of Chongqing City, significant at the 0.1 level. Through ecological urban planning, urban green space planning, demarcation of ecological redlines, and the implementation of land and industrial policies, the rationality and scientificity of the urban green space layout structure were enhanced; simultaneously, control measures and incentives positively guided and promoted the evolution of urban green spaces, with increasing constraints on the expansion of construction lands. In recent years, Chongqing’s policies on urban green space system planning, ecological redline demarcation, and land use structure adjustment have significantly influenced the scale, spatial gradient, and evolution pattern of urban green spaces. Public investment had a significant positive impact on Chongqing’s urban green spaces at the 0.05 level. Increased public investment provided substantial economic support for the construction and management of urban green spaces, enhancing the quantity and quality of urban green spaces, gradually turning the main city and its surrounding regions into major transition areas for the increase in urban green spaces, receiving certain protection and enhancement.

## 5 Discussion

The study, leveraging an integrated approach of GIS spatial analysis and mathematical statistics, reveals that the continuous outward expansion of urban construction in Chongqing has inevitably caused damage to urban green spaces. Specifically, suburban areas, which are key development sectors during this period, have witnessed the most severe encroachment, with substantial urban green spaces being converted into construction sites. Conversely, central urban districts have experienced a slight increase in urban green spaces thanks to focused ecological conservation efforts and initiatives such as “filling in the gaps with greenery.” However, these increases predominantly exist in the form of parks and roadside greenery. This research aligns to a certain extent with other studies on urban green spaces in comparably developed cities within China, reflecting a commonality in the developmental trajectories regarding urban construction and urban green space across different regions. Urban expansion generally erodes and damages urban green spaces, and integrating urban green space planning within urban spatial planning is beneficial for sustainable, efficient, and rational urban development. Notably, cities such as Sydney and New York have included landscape accessibility as an urban metric, proposing that “residents should reach a pathway to a park within 3 min” and “ensuring that everyone lives within a 10 min walk of a park,” respectively, thus incorporating perceptible ecological environment indicators into planning is becoming an important aspect of future urban green space planning and construction.

Overall, the evolution of urban green spaces in Chongqing is closely associated with the city’s development stage. Although Chongqing has gradually entered the post-industrial era, it remains in a phase of rapid development. Economic growth and industrial restructuring guide the peripheral expansion of urban construction into new and development zones, with their negative impacts still predominating. Thus, alongside continued advancements in industrial structure upgrades, there is a need to reasonably guide industrial distribution to control the sprawl of construction spaces, or to supplement urban green spaces appropriately through natural restoration, reclamation of degraded lands, and repurposing of demolished spaces, thereby enhancing ecological constraints and mitigating the negative impacts of economic factors. Despite the relatively insignificant role of social growth forces, particularly demand desires, during this period, addressing the pressures on urban green spaces from the city’s growing permanent population and ensuring an ecological, green, and healthy living environment for residents remain critical issues. Effective measures include guiding urban population flows, controlling population size, optimizing urban green space patterns, and enhancing the social equity of urban green space services. Additionally, the ecological service functions of urban green spaces are closely related to community structures and landscape distribution patterns; hence, simple increases in urban green space are insufficient. Instead, a coordinated integration of existing parks, river and lake wetlands, and other green areas into a “point-line-plane” network is essential for building a urban green space network system that better serves ecological functions.

The role of the Chongqing municipal government in overseeing urban green spaces has become increasingly pronounced, underscoring a growing focus on the management of these vital areas. The implementation of relevant policies and frameworks has actively supported the enhancement of urban green spaces. Research highlights the importance of converting strategic planning into actionable policies that steer specific initiatives, showcasing the pivotal role governmental management plays in the development of urban green spaces.

Drawing insights from the United Kingdom’s approach to urban green space development, it is clear that robust control systems, stringent policy regulations, and effective implementation mechanisms are essential for cultivating high-quality urban green environments. Therefore, the influence of governmental oversight is indispensable in the management of urban green spaces, and there is substantial potential to amplify its guiding role. Future strategies in Chongqing should focus on reinforcing government regulatory guidance, refining planning management systems, and establishing multi-tiered, tailored, and functionally organized urban green space planning and control frameworks, accompanied by robust assessment and feedback mechanisms. Additionally, the formulation of specific, transparent urban green space protection incentives, along with reward and preservation strategies, is crucial to broaden management approaches and enhance the positive impact on urban green spaces.

The study meticulously analyses the dynamic evolution of urban green space patterns based on land use classifications, quantitatively and spatially documenting the transformations in these spaces. Despite the inherent limitations in data accuracy, such as potential errors in remote sensing imagery and its interpretation, the research credibly portrays the overall trends and fulfills the intended research objectives. Administrative adjustments within Chongqing during the study period were carefully considered to ensure data consistency and relevance. While comprehensive analyses of the size and configuration of urban green spaces have been conducted, the connectivity and fragmentation of these spaces remain understudied, pointing to areas for future research enhancements.

## 6 Conclusion

This study has examined the relationship between economic factors and urban green space configurations in Chongqing during its rapid urbanization. Findings indicate that while green spaces are expanding from the suburbs into more central urban areas under the “Green Chongqing” strategy, overall green space has diminished due to urban expansion driven by population growth and construction. This reduction is especially pronounced along key city axes, with construction encroaching on previously green suburban areas. The contraction pattern of urban green spaces primarily manifests as edge erosion in main and inner suburban areas, with more severe inward contractions in central urban zones. While there have been localized increases in green spaces, these are largely restricted to specific areas, evolving from suburban green belts and ecological corridors to more integrated urban green spaces within central and edge areas of inner suburbs. Economic drivers emerge as the predominant influence on the changes in urban green spaces, with industrial development and economic expansion increasing the demand for construction land, thereby undermining the quality and extent of these green areas. Conversely, governmental regulatory measures have played a crucial role in supporting the preservation and development of urban green spaces. As environmental concerns and ecological impacts gain prominence, governmental adjustments in development strategies, along with supportive policies and investments, have significantly bolstered urban green space protection. However, the influence of corporate and residential behaviors on these spaces remains minimal, indicating a weaker impact from societal progress on economic activities and urban development patterns. In conclusion, the study underscores the need for ongoing governmental intervention to balance economic growth with environmental sustainability, ensuring the preservation and expansion of urban green spaces in Chongqing.

## Data Availability

The original contributions presented in the study are included in the article/supplementary material, further inquiries can be directed to the corresponding author.
